# The Response of *Rhodotorula mucilaginosa* to Patulin Based on Lysine Crotonylation

**DOI:** 10.3389/fmicb.2018.02025

**Published:** 2018-09-03

**Authors:** Qiya Yang, Yulin Li, Maurice T. Apaliya, Xiangfeng Zheng, Boateng N. A. Serwah, Xiaoyun Zhang, Hongyin Zhang

**Affiliations:** ^1^School of Food and Biological Engineering, Jiangsu University, Zhenjiang, China; ^2^Hubei Key Laboratory of Edible Wild Plants Conservation and Utilization, Hubei Normal University, Huangshi, China

**Keywords:** *Rhodotorula mucilaginosa*, patulin, post-translational modification, histone lysine crotonylation, degradation

## Abstract

Patulin (PAT) is a mycotoxin produced by some *Penicillium, Aspergillus*, and *Byssochlamys* species. *Rhodotorula mucilaginosa* is able to degrade PAT *in vivo* as well as *in vitro*, up till date, the process and molecular mechanism(s) involved patulin degradation still remains unknown. Protein lysine crotonylation (Kcr) plays an important role in regulating chromatin dynamics, gene expression, and metabolic pathways in mammals and eukaryotes. Investigation of the Kcr changes accompanying degradation of patulin in *R. mucilaginosa* were observed to investigate the mechanisms of patulin inhibition. Tandem mass tag (TMT) labeling and Kcro affinity enrichment, followed by high-resolution LC-MS/MS analysis, were used to perform quantitative lysine crotonylome analysis on *R. mucilaginosa*. Consequently, 1691 lysine crotonylation sites in 629 protein groups were identified, among which we quantified 1457 sites in 562 proteins. Among the quantified proteins, 79 and 46 crotonylated proteins were up-regulated and down-regulated, respectively. The differentially up expressed modified proteins were mainly involved in tricarboxylic acid cycle and gluconeogenic pathway. The differentially down expressed Kcr proteins were mainly classified to ribosome and carbohydrate transport and metabolism. Bioinformatic analyses were performed to annotate the quantifiable lysine crotonylated targets. Moreover, interaction networks and high confidence domain architectures of crotonylated proteins were investigated with the aid of bioinformatic tools, and these results showed that there was an increase in the number of yeasts with crotonylated proteins. The results also provided information on the various roles of crotonylation, which are involved in PAT degradation.

## Introduction

Patulin (PAT) is a mycotoxin produced by some *Penicillium, Aspergillus*, and *Byssochlamys* species (Sommer et al., 1974; Paster et al., 1995). It is dangerous and lethal to human beings owing to its toxicity. The primary target organs of PAT are the kidney, liver, immune system, and gastrointestinal tract. This mycotoxin is placed in group 3 by the International Agency for Research on Cancer (Patulin, 1986). PAT contamination poses a major risk to mammals; therefore, regulations have been established worldwide, with specific tolerable limits for PAT contamination in fruit-based products and juices at 50 μg kg^-1^ for adults and at 10 μg kg^-1^ for children by the European Union (Commission Regulation [EC], 2006).

A number of studies have reported that the antagonist yeasts play an important role in the degradation and/or detoxification of PAT *in vivo* and *in vitro* (Castoria et al., 2011; Dong et al., 2015; Yang et al., 2015; Mahunu et al., 2016; Zheng et al., 2016). *Pichia ohmeri* 158 produced extracellular compounds during metabolism (Coelho et al., 2007). Zheng et al. (2016) indicated that intracellular as well as extracellular enzymes produced by *Pichia caribbica* through PAT induction have the ability to degrade PAT. Furthermore, yeast cells can degrade PAT into other compounds. For instance, *Rhodosporidium kratochvilovae* strain LS11, a basidiomycete yeast, degraded PAT into desoxypatulinic acid (DPA) as the final product, while (E)- ascladiol and (Z)-ascladiol were transient products (Castoria et al., 2011).

Patulin can cause an unbalanced redox state in cells through the accumulation of reactive oxygen species (ROS), which plays a crucial role in the adaptation processes (Papp et al., 2012, 2016). Research demonstrated that treatment with 500 μM PAT significantly increased the specific activities of Cu/Zn superoxide dismutase, catalase, and glutathione *S*-transferase in order to protect the cells against the ROS-induced unbalanced redox state (Papp et al., 2012). Although several physiological and biochemical studies have attempted to explain the underlying mechanism by which yeast cause PAT degradation, the molecular mechanisms remain unclear. Thus, there is a need to extend these investigations to a greater number of proteins or genes, as well as to determine the specific processes of PAT degradation. Zheng et al. (2016) performed proteomic analyses and reported that *P. caribbica* treated with PAT led to an up-regulation of proteins involved in metabolism and stress response processes. Moreover, some genes involved in protein synthesis and modification were identified including transcription, RNA processing, translation, protein phosphorylation, and biosynthesis of amino acids were identified (Ianiri et al., 2016). Recently, there is a lack of qualitative information about the effects of postharvest biocontrol yeasts on actual PAT degradation. For example, inhibition of protein, RNA, and DNA syntheses occurs immediately after incubating PAT with yeast.

Histone post-translational modifications (PTMs) play an important role in diverse processes, such as organismal development, cell differentiation, cell death, and also contribute to cancer development due to epigenetic deregulation (Berdasco and Esteller, 2010; Tan et al., 2011). There are many types of histone PTMs including methylation, acetylation, phosphorylation, and histone ubiquitination (Zhao et al., 2010; Tan et al., 2011; Zhang et al., 2011; Olsen and Mann, 2013; Weinert et al., 2013; Liu and Gao, 2016; Mohibi et al., 2016; Nishiyama et al., 2016; Penard-Lacronique and Bernard, 2016). In recent years, histone lysine crotonylation (Kcr), a new PTM has been discovered. Tan et al. (2011) investigated histone Kcr, which is different from histone lysine acetylation (Kac) in structure and function and found that histone Kcr marks potential enhancers and activates genes expressions in human somatic and mouse male germ cell genomes. Moreover, they found histone Kcr on sex chromosomes and specific genes in post-meiotic male germ cells. Histone Kcr marks post-meiotically expressed X-linked genes and likely affects the chromatin structure, thereby facilitating histone replacement. Montellier et al. (2012) studied the molecular basis of the epigenetic shaping of haploid male germ cells and found that histone Kcr contributes to the maintenance of male haploid cell genes in the active state and is associated with post-meiotic phases of spermatogenesis. Bao et al. (2014) found that Sirt1, Sirt2, and Sirt3 can catalyze the hydrolysis of lysine crotonylated histone peptides and proteins. More importantly, Sirt3 functions as a decrotonylase to regulate histone Kcr dynamics and gene transcription in living cells. Xiong et al. (2016) demonstrated a new regulatory mechanism of histone crotonylation recognized by double plant homeodomain finger (DPF) family members, which accommodated a wide range of histone Kcr. Crystal structures of the DPF domain of monocytic leukaemia zinc-finger protein (MOZ) in complex with H3K14 crotonylation (H3K14cr), H3K14 butyrylation (H3K14bu), and H3K14 propionylation (H3K14pr) peptides revealed that these non-acetyl acylations are anchored in a hydrophobic ‘dead-end’ pocket with selectivity for crotonylation that arises from intimate encapsulation and an amide-sensing hydrogen bonding network. Additionally, AF9 YEATS, which co-localizes with crotonylated histone H3, positively regulates gene expression in yeast in a domain-dependent manner. By using a cell-based model, they defined the evolutionarily conserved yeast domains as a family of crotonyl lysine readers and specifically demonstrated that the YEATS domain of AF9 directly links histone crotonylation to active transcription (Li et al., 2016).

*Rhodotorula mucilaginosa* was reported to degrade PAT (Yang et al., 2015); however, the cellular enzymes and proteomes that regulate the degradation of PAT remain unknown. Zheng et al. (2017) had researched patulin exposure prompt *R. mucilaginosa* to produce a series of actions to resist or degrade patulin, including Kac, they found the Kac level of pyruvate carboxylase and citrate synthase which were the key enzyme of TCA cycle were up-regulated proteins by the stress caused by pLINatulin. Wei et al. (2017) had found that histone crotonylation is not redundant to histone acetylation but is broadly essential for transcription and also supports potential roles of histone crotonylation in other chromatin-associated events. Investigation of the Kcr changes accompanying degradation of patulin in *R. mucilaginosa* were observed to investigate the mechanisms of patulin inhibition. The aim of this study was to use an integrated approach that involved TMT labeling, HPLC fractionation, Kcr antibody affinity enrichment, and LC-MS/MS to quantify the dynamic changes in the complete crotonylome in *R. mucilaginosa*. In this study, we evaluated the proteins that play a crucial role in the response to PAT treatment, analyzed the crosstalk and protein-protein interaction (PPI) network among the various Kcrs, and predicted the Kcr pathway that may be involved in PAT degradation.

## Materials and Methods

### Yeast

*Rhodotorula mucilaginosa* was isolated from the surface of peach orchard (Zhenjiang, Jiangsu Province). *R. mucilaginosa* isolates were prepared immediately prior to their use, as described by Yang et al. (2015) with slight modifications. Yeast cell pellets were suspended in sterile distilled water and adjusted with a hemocytometer to 1 × 10^8^ cells/mL. Suspensions of *R. mucilaginosa* were added to 250 mL Erlenmeyer flasks containing 50 mL of nutrient yeast dextrose broth (NYDB) medium and either the cell suspension of *R. mucilaginosa* (1 × 10^8^ cells/mL) alone or the cell suspension of *R. mucilaginosa* (1 × 10^8^ cells/mL) supplemented with 5 μg/mL patulin. The flasks were then incubated at 28°C for 15 h in a rotary shaker at 180 rpm. Following incubation, cells were centrifuged at 7000 × *g* for 10 min and the cells were harvested and freeze-dried for subsequent experiments.

### Protein Extraction and Trypsin Digestion

In this experiment, the protein extraction and trypsin digestion were performed according to Hao et al. (2017) and Wang et al. (2017) with slight modifications. The protein was redissolved in buffer [8 M urea, 100 mM tetraethylammonium bromide (TEAB), pH 8.0] and the concentration was determined with a 2-D Quant kit (GE Healthcare, Buckinghamshire, United Kingdom) according to the manufacturer’s instructions.

For digestion, the protein solution was reduced with 10 mM DTT for 1 h at 37°C and alkylated with 20 mM iodoacetamide (IAA) for 45 min at room temperature in the dark. For the trypsin digestion, the protein sample was diluted by adding 100 mM TEAB to urea at a concentration less than 2 M. Finally, trypsin was added to the protein in a 1:50 ratio for the first digestion overnight and a 1:100 mass ratio for a second 4 h digestion. Approximately 100 μg of protein for each sample was digested with trypsin for the following experiments.

### TMT Labeling

In this experiment, the method of protein extraction and trypsin digestion was carried out according to the method described by Wang et al. (2017) with some modifications. Briefly, one unit of TMT reagent (defined as the amount of reagent required to label 1 mg of protein) was thawed and reconstituted in 24 μL acetonitrile (ACN). The peptide mixtures were then incubated for 2 h at room temperature and pooled, desalted and dried by vacuum centrifugation.

### Affinity Enrichment

In this experiment, the method of affinity enrichment was carried out according to the method described by Zheng et al. (2017) with some modifications. The bound peptides were eluted from the beads with 0.1% trifluoroacetic acid. The eluted fractions were combined and vacuum- dried. The resultant peptides were cleaned with C_18_ ZipTips (Millipore) according to the manufacturer’s instructions and were used in the LC-MS/MS analysis.

### LC-MS/MS Analysis

The methods of protein extraction and trypsin digestion were performed according to the protocol reported in Sun et al. (2017) with slight modifications. The gradient was comprised of an increase from 6 to 22% of solvent B (0.1% FA in 98% ACN) for 40 min, from 22 to 35% over 12 min, climbing to 80% over 4 min, and then holding at 80% for the last 4 min. A constant flow rate of 300 nL/min was maintained on an EASY-nLC 1000 UPLC system, and the resulting peptides were analyzed by a Q Exactive^TM^ plus hybrid quadrupole-Orbitrap mass spectrometer (Thermo Fisher Scientific).

The peptides were subjected to Nanospray ionization (NSI) source followed by tandem mass spectrometry (MS/MS) in Q Exactive^TM^ plus (Thermo) coupled with an online Ultra Performance Liquid Chromatography (UPLC). For MS scans, the m/z scan range was 350 to 1800. Fixed first mass was set as 100 m/z.

### Database Search

The resultant MS/MS data were processed using MaxQuant with the integrated Andromeda search engine (v.1.5.2.8). Tandem mass spectra were searched against the 6010.fasta database concatenated with a reverse decoy database. False discovery rate thresholds for proteins, peptides and modification sites were specified at 1%. The minimum peptide length was set at seven amino acids. For quantification, TMT-6-plex was selected. All the other parameters in MaxQuant were set to the default values. The site localization probability was set as >0.75.

### Motif Analysis

Motif-x software was used to analyze the model of sequences that constituted amino acids at specific positions of modify-21-mers (10 amino acids upstream and downstream of the site) in all protein sequences. All the database protein sequences were used as background database parameters, while other parameters were set to their defaults.

### Bioinformatic Methods

#### GO Annotation

The GO annotation proteome was obtained from the UniProt-GOA database^1^. Identified the protein IDs were converted to UniProt IDs and then mapped to GO IDs.

#### Enrichment of Protein Domain Analysis

Correction for multiple hypothesis testing was carried out using standard false discovery rate control methods and domains with a corrected *p*-value < 0.05 were considered significant.

#### PPI Analysis

To generate the PPI network, the identified proteins were queried against the STRING database (v10.0) for PPIs. Only the interactions between the proteins that belong to the searched data set were selected, thereby excluding external proteins. Interactions that have a STRING high confidence score were fetched (score ≥ 0.9 without ‘text-mining evidence,’ see STRING instructions for details). CytoScape (v3.3.0) was applied to visualize the interaction network generated from STRING.

## Results

### Overview of Quantitative Analysis

Altogether, 1691 crotonylation sites in 629 proteins were identified, among which 1457 crotonylation sites in 562 proteins were quantified (**Table 1**). In this study, the levels of proteins that showed quantitative ratios greater than 1.2 or less than 0.83 were considered up-regulated or down-regulated, respectively. The differentially quantified sites and proteins are summarized in **Table 2**. Up-regulation was observed for 102 crotonylation sites in 79 proteins, while 67 crotonylation sites in 46 proteins were down-regulated.

**Table 1:** Summary of identified and quantified sites and proteins.

Name	Identified	Quantified
Sites	1691	1457
Proteins	629	562

**Table 2:** Summary of differentially quantified sites and proteins.

Name		Up-regulated (>1.2)	Down-regulated (<1/1.2)
Yeast+PAT/Yeast	Proteins	79	46
	sites	102	67

### Functional Classification of Differentially Expressed Proteins

#### Gene Ontology Classification

To understand the possible roles of the Kcr, functional classification of the identified proteins was performed. To further understand the distribution of identified proteins, information on GO annotation is summed up in accordance with each quantifiable level 2 protein GO term in **Tables 3, 4**. These tables present a functional view of the up-regulated and down-regulated proteins according to their categories such as cellular component, molecular function, and biological process. Among the assigned processes, the top three categories were metabolic process, cellular process, and single-organism process with up-regulated or down-regulated proteins. The three functions (catalytic activity, binding, and structural molecular activity) were the most important molecular functions for either up-regulated or down-regulated proteins. The GO analysis suggested that the crotonylated proteins had a wide range of biological processes and molecular functions in *R. mucilaginosa*.

**Table 3:** The distribution of up-regulated proteins in GO terms of level 2 (Y+PAT/Y).

GO terms Level 1	GO terms Level 2	Number of protein
Biological process	Metabolic process	48
	Cellular process	42
	Single-organism process	31
	Localization	8
	Biological regulation	4
	Cellular component organization or biogenesis	4
	Other	2
Cellular component	Cell	26
	Organelle	18
	Macromolecular complex	16
	Membrane	8
Molecular function	Catalytic activity	38
	Binding	35
	Structural molecule activity	11
	Transporter activity	4
	Antioxidant activity	2
	Molecular function regulator	2

**Table 4:** The distribution of down-regulated proteins in GO terms of level 2 (Y+PAT/Y).

GO terms Level 1	GO terms Level 2	Number of protein
Biological process	Metabolic process	24
	Cellular process	18
	Single-organism process	15
	Localization	3
	Response to stimulus	2
	Other	1
Cellular component	Cell	10
	Organelle	7
	Macromolecular complex	6
	Membrane	2
Molecular function	Binding	23
	Catalytic activity	22
	Structural molecule activity	5
	Transporter activity	4
	Electron carrier activity	3
	Other	1

#### Classification of Subcellular Location

**FIGURE 1:**
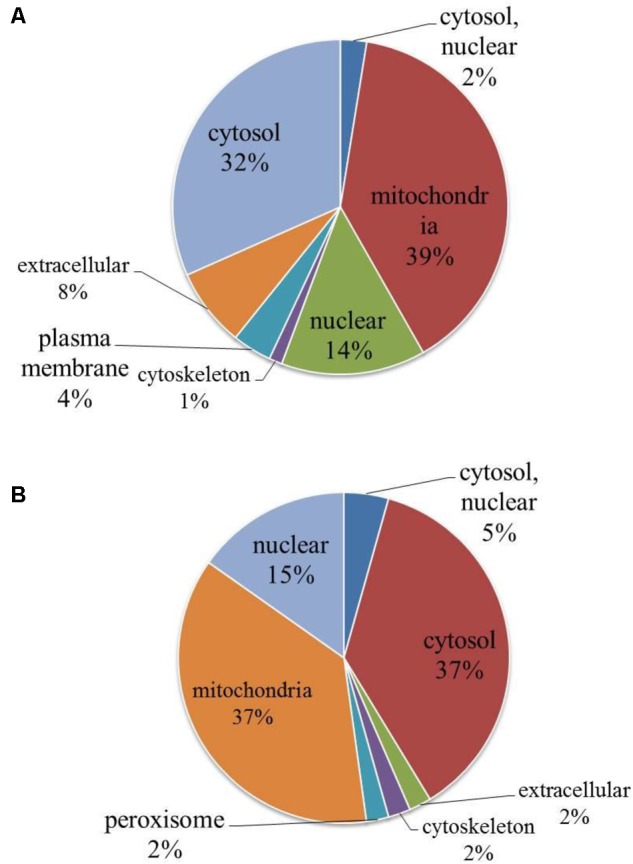
The subcellular up-regulated and down-regulated proteins (Y+PAT/Y). **(A)** Subcellular localization of the up regulated proteins by patulin. **(B)** Subcellular localization of the down regulated proteins by patulin.

Based on the results of the subcellular location annotation of the identified proteins, we classified the quantifiable proteins into each subcellular location (**Figure 1**). The results indicated that the largest group of up-regulated proteins was associated with the mitochondria (39%), and the other two subcellular components were associated with the cytosol (32%) and the nucleus (14%) (**Figure 1A**). The overall trends in subcellular localization were similar in down-regulated proteins with the cytosol (37%), mitochondria (37%), nucleus (15%) among the subcellular components (**Figure 1B**).

#### Motif Analysis

**FIGURE 2:**
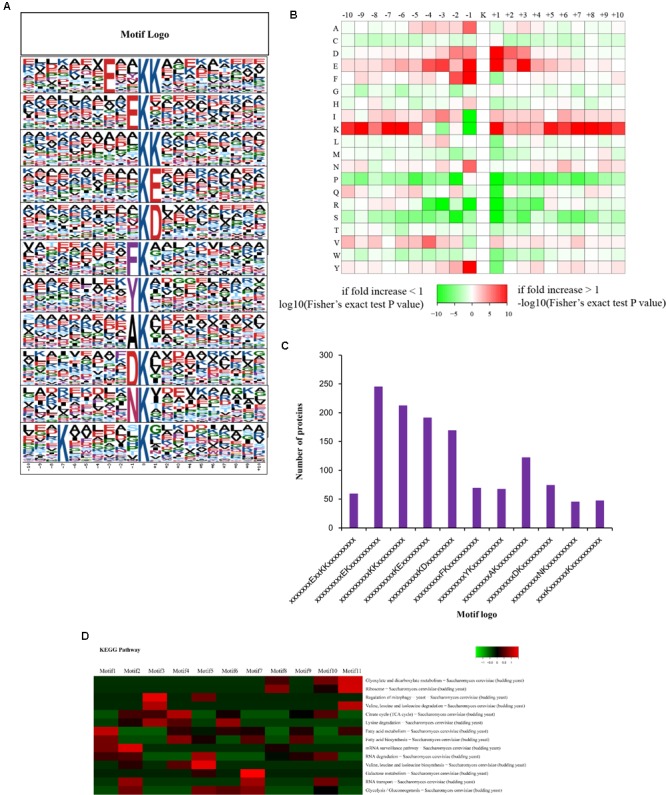
Analysis the characters of the identified peptides. **(A)** Crotonylation motifs were constructed with Motif-X software. The central K (at position 0) indicates the crotonylation lysine. All the surrounding amino acid residues are indicated with the letters in different heights which is consistent with their frequencies in respective positions. **(B)** Heat map showing the frequency of the amino acid residues around the crotonylated site based on analysis of all the identified peptides. **(C)** Number of the crotonylated peptides in each motif. **(D)** Heat map showing the enriched motifs in the representative KEGG pathways.

The motif-x program was used to extract enriched motifs from the set of sequences. The peptides were discovered to contain 20 amino acid residues that surround the crotonylation lysine (from positions -10 to +10), and these motifs were illustrated as EXXKcrKcr, EKcr, KcrKcr, KcrE, KcrD, FKcr, YKcr, AKcr, DKcr, NKcr, and KcrXXXXXXKcr. Kcr is the crotonylation at lysine, and X represents a random amino acid residue (**Figure 2A**). A survey of these motifs suggested that several kinds of amino acid residues are found within these motifs. These conserved amino acid residues include various kinds of amino acid including glutamate (E), aspartate (D), phenylalanine (F), tyrosine (Y), alanine (A), and asparagine (N) (**Figure 2B**). As illustrated in **Figure 2B**, these motifs can be sorted into four categories: (i) position +1 is an aspartate residue, (ii) position -1 is a tyrosine or a phenylalanine residue, (iii) position +3, +1, or -1 is a glutamate residue, (iv) position +10, +9, +8, +7, +5, +1, -6, -7, -9, or -10 is a lysine residue. These results suggest that the crotonylated system prefers to modify the lysine residue in order to be surrounded with negatively charged residues from -10 to +10. All the motifs varied in their abundance in the crotonylated peptides, and motif 2 was the most extensively distributed motif (**Figure 2C**). Enrichment analysis indicated that seven motifs exhibited different distributions over the KEGG pathway (**Figure 2D**). Motif 2, motif 3, motif 5, and motif 7 were significantly over-represented in the mRNA surveillance pathway, in the regulation of mitophagy, in valine, leucine, and isoleucine biosynthesis and in galactose metabolism, respectively. However, motif 11 was enriched in three pathways (glyoxylate and dicarboxylate metabolism, ribosome degradation, and valine, leucine, and isoleucine degradation).

### Functional Enrichment of Differentially Quantified Proteins

#### GO Enrichment

**FIGURE 3:**
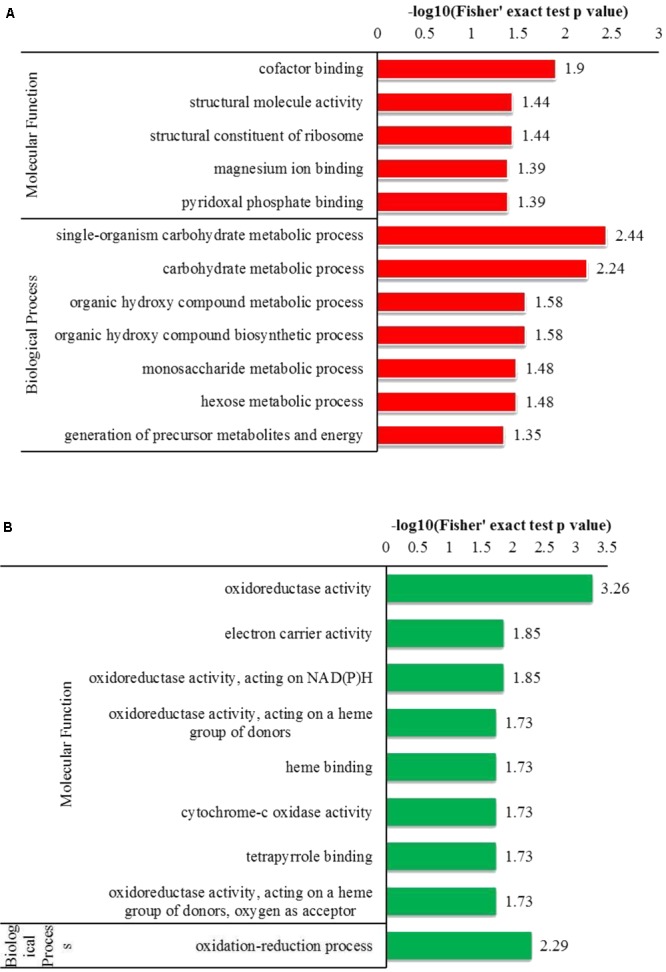
GO-based enrichment analysis of up-regulated and down-regulated proteins (Y+PAT/Y). **(A)** Classification of the up-regulated proteins with molecular function and biological process. **(B)** Classification of the down-regulated proteins with molecular function and biological process.

To determine the preferred protein types for Kcr, GO enrichment analysis was performed on the crotonylation data (**Figure 3**). Among the identified proteins, 465 crotonylated proteins were annotated for their biological process, and 366 for their corresponding molecular functions. Based on the biological process, the enrichment analysis of the identified proteins showed that proteins related to single-organism carbohydrate metabolism were high for up-regulated proteins, while the oxidation-reduction process was significant for down-regulated proteins.

#### Domain Enrichment

**FIGURE 4:**
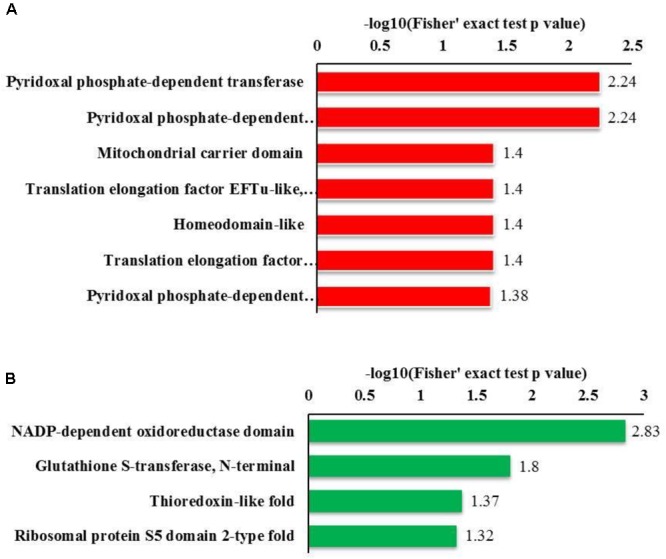
Bioinformatics analysis of the lysine crotonylated proteins of *R. mucilaginosa* (Y+PAT/Y). **(A)** Classification of the down regulated lysine crotonylated proteins with molecular function and biological process. **(B)** Classification of the up regulated lysine crotonylated proteins with molecular function and biological process.

Regarding the GO enrichment of domains, the analysis revealed that Kcr substrates were abundant in pyridoxal phosphate-dependent transferase. The other substrates that were abundant included subdomain 1 in up-regulated proteins and the NADP-dependent oxido-reductase domain and glutathione *S*-transferase (N-terminal) in down-regulated proteins (**Figure 4**). Overall, our data suggest that mitochondrial and cytosolic processes may be strictly regulated by crotonylation.

#### Interaction Network of Crotonylated Proteins

**FIGURE 5:**
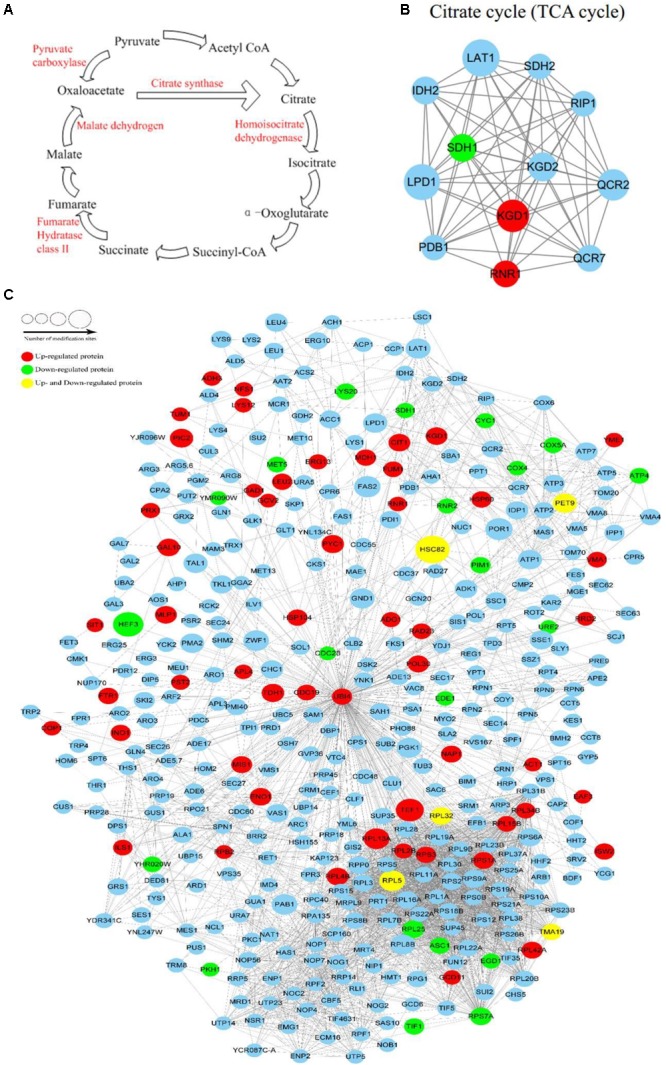
The metabolic pathway influenced by patulin and Protein interaction network of Kcr proteins. **(A)** Lysine crotonylation of metabolic enzymes identified by MS based proteomics in TCA cycle. **(B)** Protein interaction network of TCA cycle. **(C)** Protein interaction network of Kcr proteins. Size of the node represent different number of modification sites. Different colors represent differential regulated type: up-regulated protein (red); down-regulated protein (green); up-regulated and down-regulated protein (yellow); no differential expressed protein (light blue).

The pathway analysis revealed that proteins with lysine crotonylated ions were involved in multiple biological processes, including the TCA cycle, glycolysis, and amino acids synthesis. There were several Kcr sites involved in TCA metabolic processes, such as pyruvate carboxylase, citrate synthase, homoisocitrate dehydrogenase, malate dehydrogenase, and fumarate hydratase (class II). These sites were up-regulated due to the stress caused by PAT (**Figure 5A**). The interaction networks of crotonylated proteins involved in the TCA cycle are shown in **Figure 5B**. Three up-regulated Kcr sites (pyruvate kinase, glyceraldehyde-3-phosphate dehydrogenase, and enolase) were involved in glycolysis. The PPIs for the identified crotonylated proteins revealed an interaction network that consisted of 408 direct physical interactions. This suggested that the crotonylated proteins were associated with a wide range of protein interactions. The number of crotonylation sites was represented for each interaction network (**Figure 5C**). The degree of the node was an important parameter to evaluate the correlation of a protein network. Crotonylated protein UBI4 showed the highest degree among the proteins in the network, followed by RPS3, RPL3, RPS5, and UBI4 proteins.

## Discussion

Prior molecular studies clearly explain the complex mechanisms involved in PAT degradation and/or detoxification. In a previous study, unique genes were identified to degrade PAT in the model basidiomycete yeast *Sporobolomyces* (Ianiri et al., 2016). Proteomic profiling of protein Kcr was identified in human somatic and mouse male germ cell genomes (Tan et al., 2011). Wu et al. (2017) found that lysine crotonylation participates in a wide range of biological functions and processes, both the crotonylation and acetylation levels of most core histones sites and a number of non-histone proteins as well as some known substrates of class IIa and IIb HDACs were up-regulated after SAHA treatment. These results suggest that SAHA may have decrotonylation inhibitory activities on both histones and non-histone proteins by inhibiting HDACs. Although much is known about the currently identified histone Kcr and its functions in humans and mice, information about the functions of Kcr in yeast cells and its potential role in signaling during stress caused by PAT in yeast cells remains unclear. Bioinformatic tools were used to analyze the association of protein Kcr with cellular processes, metabolic pathways, and protein interactions.

The classification of the biological processes showed that the largest protein group is composed of enzymatic proteins that are associated with metabolic processes, which included both up-regulated and down-regulated proteins. Binding and catalytic activity were the dominant molecular functions of the up- and down-regulated proteins.

Riley and Showker (1991) found that the basic toxic effects of PAT were on plasma membranes and non-protein sulfhydryls (glutathione) in LLC-PK_1_ cells. Their results revealed that the endoplasmic reticulum calcium transport ATPase is a sensitive target for cyclopiazonic acid as well as the antioxidant activity of indole tetramic acids, which are serially connected but occur parallel to the depletion of sulfhydryls, an increase in ^86^Rb^+^ efflux, dome collapse, and eventually the loss of cell viability. Kim et al. (2010) used natural phenolic chemo-sensitizing agents to overcome fludioxonil resistance in *Penicillium expansum*, and found that fungal mitochondrial superoxide dismutase (Mn-SOD) plays a role in protecting cells from oxidative damage against alkyl gallates. Our results demonstrated that Mn-SOD was up-regulated in the lysine crotonylation levels, which suggested that the antioxidant response was activated in *R. mucilaginosa* cells against PAT. Enolase (Eno) plays an important role in transcription, apoptotic regulation, and cell differentiation. Eno is a phosphopyruvate hydratase that is considered to be a metallo-enzyme responsible for the catalysis or inter-conversion of 2-phosphoglycerate to phosphoenolpyruvate. Eno was down-regulated when *P. expansum* was exposed to nitric oxide; this disrupted glycolysis and subsequently influenced the Krebs cycle (Subramanian and Miller, 2000; Lai et al., 2014). In our results, we found up-regulation of Eno among the lysine crotonylation levels, indicating that PAT may have stimulated glyco-metabolism of *R. mucilaginosa*. Additionally, glycolysis of yeast cells was obstructed, thereby improving Eno activity. Ismaiel and Papenbrock (2014) found that maize root tip cells treated with 25 μg/mL PAT showed rumpled morphological changes in the cytoplasm and cytoplasmic organelles by using transmission electron microscopy. Cytoplasmic dissolution could be caused by the biochemical basis of PAT toxicity that involves respiratory inhibition. The present results demonstrated higher levels of cytoplasmic proteins with Kcr and confirmed that PAT could cause deterioration in cytoplasmic organelles. The changes in the cyto-morphological modification showed that *R. mucilaginosa* cells were stimulated by PAT.

Zhang et al. (2015) indicated that PAT caused an increase in ROS and a loss of SOD and glutathione (GSH) activities, which resulted in oxidative stress. These findings suggested that PAT might induce apoptosis in HEK293 cells through oxidative stress and damaged kidney cells. Moreover, PAT was found to cause rat hepatocyte cell damage by the formation of adducts with thiol-containing proteins in cellular components, such as GSH and cysteine-containing proteins. Simultaneous suppression of gap junction-mediated intercellular communication (GJIC) and GSH depletion initiates ROS generation, followed by mitochondrial membrane depolarization that causes a simultaneous increase in [Ca^2+^]_i_ and cytoplasmic acidification, which leads to depolarization of the plasma membrane (Harwig et al., 1973; Barhoumi and Burghardt, 1996). There are many enzymes with a sulfhydryl group in their active sites that are sensitive to PAT (Moake et al., 2005), including Na^+^-K^+^- dependent ATPase (Phillips and Hayes, 1977, 1978; Riley and Showker, 1991), muscle aldolase (Ashoor and Chu, 1973), aminoacyl-tRNA synthetase (Arafat et al., 1985), and RNA polymerase (Moulé and François, 1977). Most heat shock proteins (HSPs) belong to a well-conserved family of cellular proteins that are responsible for maintaining the stability of other proteins within the cells, and for protecting proteins from damage due to environmental stress. Mitochondrial matrix HSPs, are typically responsible for pro-survival and pro-apoptotic functions, as well as the transportation and refolding of proteins from the cytoplasm into the mitochondrial matrix. HSPs have evolved as intracellular protein-folding molecules to protect newly synthesized polypeptides from misfolding, prevent improper interactions with other molecules, or mediate conformational changes of individual proteins and formation of multimeric complexes (Gullo and Teoh, 2004; Chandra et al., 2007; Kim et al., 2009). Based on 2-DE and MS analyses, Lai et al. (2014) identified that HSP70 was up-regulated and HSP60 was down-regulated in response to exogenous NO in *P. expansum*. These results suggested that NO might impair the synthesis and transportation of some mitochondrial proteins that are essential for cell growth. HSP60 and HSP70 are responsive to environmental stresses, including NO stress. In our results, HSPs were found to be up-regulated following PAT treatment, suggesting PAT treatment led to oxidative stress in *R. mucilaginosa* cells. Defense response by HSP activity can stimulate resistance to PAT toxicity and expel PAT from the yeast cells.

Moreover, PAT induced intra- and inter-molecular protein crosslinking *in vitro*. It was found that cysteine thiol group was preferred for PAT-mediated crosslinking reactions, but lysine and histidine side chains and α-amino groups also exhibited reactivity. PAT can act as a homobifunctional as well as a heterobifunctional crosslinking agent (Fliege and Metzler, 1999). Zhang et al. (2016) reported that the YEATS domain of AF9 preferably binds croton-lysine over acetyl-lysine in histone H3. Croton-lysine of histone H3 (lysine 18) was deeply engulfed in an aromatic cage of the YEATS domain where the carbonyl oxygen of croton-lysine forms a hydrogen bond with the amide backbone of the protein residue Tyr78. Their findings presented a new structural mechanism of protein–protein interactions mediated by histone Kcr, and showed the mechanism by which cells interpret acyl-lysine marks in different biological contexts.

Boussabbeh et al. (2015) indicated that PAT caused ER stress and activated the unfolded protein response. ER stress is related to the induction of the mitochondrial apoptotic pathway that occurs with ROS induction, a drop in mitochondrial membrane potential, and caspase activation. In our results, the largest group of up-regulated proteins were related to ROS activity and the TCA cycle and were associated with the mitochondria. In this study, we identified five crotonylated enzymes that were found to be associated with the TCA cycle. They include pyruvate carboxylase, citrate synthase, homoisocitrate dehydrogenase, fumarate hydratase (class II), and malate dehydrogenase. These five enzymes play important roles in the central metabolic pathway.

Wei et al. (2017) had found that histone crotonylation is not redundant to histone acetylation but is broadly essential for transcription and also supports potential roles of histone crotonylation in other chromatin-associated events. Zheng et al. (2017) had researched the Kac level of pyruvate carboxylase and citrate synthase which were the key enzyme of TCA cycle were up-regulated proteins by the stress caused by patulin. They also found that ROS accumulation increase the expression levels of oxidoreductase to maintain the balance of redox state and to reduce the patulin toxicity in *R. mucilaginosa*. Huang et al. (2018) found that the most downregulated crotonylome alterations under p300 deficiency concern components of the nonsense-mediated decay, infectious disease, and viral/eukaryotic translation pathways. Moreover, some p300-targeted Kcr substrates are potentially linked to diseases such as cancer. In our results, the largest group of up-regulated protein were related to ROS activity and the TCA cycle and were associated with the mitochondria. The results suggested that the Kac and Kcr regulated the TCA metabolic process and defense to ROS in response to the stress induced by the patulin. It was interesting to find the nice correlation between promoter Kac and Kcr and the mutant might repress transcription through deacylation other than decrotonylation.

## Conclusion

Bioinformatic analysis to understand the regulation of differentially expressed proteins, transcription factors functions, and the enzymes that regulate the PAT degradation processes is extremely important for further research. However, in order to accomplish this goal using yeast, we must not only select the appropriate protein targets with specific functions but also develop antibodies specifically against those selected targets. This is important for examining the phenotypic and functional changes and to address the unanswered questions in the degradative process. Future work should be devoted to elucidate whether and how histone crotonylation readers mediate the essential function of histone crotonylation in transcription in *R. mucilaginosa* treated with patulin.

## Author Contributions

HZ designed the experiments and revised the manuscript. QY and YL performed the experiments and analyzed results. MA and XfZ provided direction in experimental methods and revised the manuscript. BS and XyZ revised the manuscript.

## Conflict of Interest Statement

The authors declare that the research was conducted in the absence of any commercial or financial relationships that could be construed as a potential conflict of interest. The reviewer GP and handling Editor declared their shared affiliation.

## Footnotes

1 http://www.ebi.ac.uk/GOA/
